# Comprehensive characterization of oscillatory signatures in a model circuit with PV- and SOM-expressing interneurons

**DOI:** 10.1007/s00422-021-00894-6

**Published:** 2021-10-09

**Authors:** Marije ter Wal, Paul H. E. Tiesinga

**Affiliations:** 1 Department of Neuroinformatics, Donders Institute, Radboud University, Heyendaalseweg 135, 6525 AJ Nijmegen, The Netherlands; 2 School of Psychology, University of Birmingham, Edgbaston, B15 2TT UK

**Keywords:** Oscillations, Neural circuits, PV-interneurons, SOM-interneurons, Clustering

## Abstract

Neural circuits contain a wide variety of interneuron types, which differ in their biophysical properties and connectivity patterns. The two most common interneuron types, parvalbumin-expressing and somatostatin-expressing cells, have been shown to be differentially involved in many cognitive functions. These cell types also show different relationships with the power and phase of oscillations in local field potentials. The mechanisms that underlie the emergence of different oscillatory rhythms in neural circuits with more than one interneuron subtype, and the roles specific interneurons play in those mechanisms, are not fully understood. Here, we present a comprehensive analysis of all possible circuit motifs and input regimes that can be achieved in circuits comprised of excitatory cells, PV-like fast-spiking interneurons and SOM-like low-threshold spiking interneurons. We identify 18 unique motifs and simulate their dynamics over a range of input strengths. Using several characteristics, such as oscillation frequency, firing rates, phase of firing and burst fraction, we cluster the resulting circuit dynamics across motifs in order to identify patterns of activity and compare these patterns to behaviors that were generated in circuits with one interneuron type. In addition to the well-known PING and ING gamma oscillations and an asynchronous state, our analysis identified three oscillatory behaviors that were generated by the three-cell-type motifs only: theta-nested gamma oscillations, stable beta oscillations and theta-locked bursting behavior, which have also been observed in experiments. Our characterization provides a map to interpret experimental activity patterns and suggests pharmacological manipulations or optogenetics approaches to validate these conclusions.

## Introduction

With the introduction of genetic tools into neuroscience, particularly optogenetics, the possibilities to study the functional roles of and relationships between different cell types in the brain have improved dramatically (Callaway 2005; Fenno et al. 2011; Luo et al. 2018). Although representing a numerical minority of all neurons, inhibitory interneurons have received most attention in recent years. Two groups of inhibitory interneurons are of particular interest: interneurons that express the calcium-binding protein parvalbumin (PV, reviewed in (Hu et al. 2014)), such as basket cells and chandelier cells, and interneurons expressing the neuropeptide somatostatin (SOM, for a review see (Urban-Ciecko and Barth 2016)), e.g., Martinotti cells in neocortex and oriens-lacunosum moleculare (O-LM) cells in hippocampus. PV and SOM cells are the two-interneuron types that most frequently occur in cortex, with PV positive cells making up about 40–50% (Markram et al. 2004; Rudy et al. 2011) and SOM cells about 30% of the interneuron population (Rudy et al. 2011). About half of the remaining share is made up of a third cell type, expressing Vasoactive Intestinal Peptide (VIP, Rudy et al. 2011). Genetic tools have made it possible to identify these interneuron types in vivo and in vitro and assess their connection profiles (for example: Pfeffer et al. 2013), and to selectively stimulate or inhibit activity through the expression of light-sensitive ion channels, namely channelrhodopsin and halorhodopsin, respectively (reviewed in: Fenno et al. 2011).

Both PV and SOM are expressed by several interneuron cell types, but despite this diversity, these groups generally have some distinct biophysical properties (Gouwens et al. 2020), which can effectively be captured by simple spiking neuron models (Billeh et al. 2020). PV cells are generally fast spiking (FS), reaching high firing rates (Kawaguchi and Kubota 1993), while SOM cells spike less often, but become active at lower input levels and are hence referred to as ‘low-threshold spiking’ (LTS). PV basket cells target the soma and basal dendrites of neighboring pyramidal cells and PV chandelier cells target the initial axonal element (Klausberger and Somogyi 2008). The axons of these cell types are highly branched, suggesting they can produce strong inhibition in the local circuit (Hu et al. 2014). Conversely, SOM cells are mostly found in the superficial layers of the cortex, mostly targeting the higher parts of the apical dendrites and tufts (Sik et al. 1995; Wang et al. 2004). As a result, SOM cell activity is associated with inhibition of NMDA-mediated calcium spikes and bursting (Spruston et al. 1995).

PV, SOM and VIP cells were also found to have distinct connection patterns with other cell types. In mouse V1, PV cells tend to inhibit pyramidal cells and each other, but not other interneuron types (Pfeffer et al. 2013). On the other hand, SOM cells do not inhibit each other, but strongly inhibit both PV and VIP expressing interneurons (Pfeffer et al. 2013). SOM cells have been shown to mediate lateral inhibition between neighboring pyramidal cells (Silberberg and Markram 2007). VIP cells were found to inhibit predominantly SOM cells, leading to disinhibition of PV and pyramidal cells, a pattern that was found in both V1 (Pfeffer et al. 2013), motor cortex (Lee et al. 2013) and auditory and prefrontal cortex (Pi et al. 2013). In prefrontal cortex, PV, but not SOM, cells were found to preferentially connect to some types of excitatory cells, while avoiding others (Lee et al. 2014a), suggesting that several connection patterns can co-exist within a cortical area.

Interneurons are likely to play many essential roles in neural function, from preventing overall runaway excitation to complex computation (Isaacson and Scanziani 2011; Roux 2015; Cardin 2019). Based on the differences in both biophysical properties and connection patterns between PV and SOM cells, it is expected that these cell types also show distinct functional activation patterns. Indeed, several studies have identified differences between PV and SOM cells in locking strength and phase preference within neural oscillations. In hippocampus, O-LM cells preferably lock to the peak of the 4–8 Hz theta oscillation, while PV cells lock to the trough (Klausberger et al. 2003; Lapray et al. 2012). O-LM cells show a marked increase in activity during theta oscillations, while PV firing is relatively low (Klausberger et al. 2003). However, rhythmic optogenetic stimulation of PV cells led to resonance in the theta frequency band (Stark et al. 2013). Interestingly, reducing inhibitory inputs onto PV cells did not affect hippocampal gamma rhythms, but did reduce theta power (Wulff et al. 2009). Furthermore, PV, but not SOM inactivation, has been shown to affect the timing of hippocampal place cells firing within the theta rhythm, while SOM inactivation lead to burst firing (Royer et al. 2012). In addition to theta-locking, PV cells are also locked to gamma oscillations nested within the theta periods. Modeling of hippocampal circuits has suggested that the generation of these theta-nested gamma oscillations depends on an interaction between PV and SOM cells (White et al. 2000; Rotstein et al. 2005; Vierling-Claassen et al. 2010; Bezaire et al. 2016), in line with the experimental work by (Wulff et al. 2009), but these models do not agree on which connection is critical and whether additional membrane dynamics are required. Other modeling work has suggested SOM cells are not required for theta generation (Ferguson et al. 2017). It has also been suggested that these different interneuron types can sustain independent slow and fast nested gamma rhythms (Keeley et al. 2017), in line with the findings from (Colgin et al. 2009). These findings therefore suggest that PV and SOM indeed play complementary roles in hippocampal circuits. The exact nature of these roles remains to be determined and may depend on the circuit activity and differ for other brain regions, stressing the need for a comprehensive study of possible circuits motifs involving PV and SOM cells.

In addition to theta oscillations, which have been related to memory formation (Colgin 2016) and periodic attentional sampling (VanRullen 2016), several other rhythms are prominently found in local field potentials: alpha frequencies (8–12 Hz) which is thought of as a sign of global inhibition (Jensen and Mazaheri 2010), and beta (12–30 Hz) and gamma frequencies (30–90 Hz) associated with anything between coding and communication of information in neural circuits, to a wide range of cognitive functions (for reviews see: Jensen et al., 2007; Spitzer & Haegens, 2017). With the exception of gamma oscillations, little is known about the generation of these rhythms (for a review see: Wang 2010). Inhibition and stimulation of SOM cells was demonstrated to reduce, respectively, enhance, visually induced beta oscillations, while PV cells were shown in be involved in both beta and gamma frequencies, both under anesthesia (Kuki et al. 2015) and in behaving animals (Chen et al. 2017). Other work has suggested that beta oscillations are independent of GABA_A_-mediated inhibition, excluding a role for PV-inhibition in beta-generation (Roopun et al. 2006). No modeling framework has so far accounted for all these findings. It has been established that PV cells are essential for the generation of gamma rhythms in hippocampus (Whittington et al. 1995; Traub et al. 2000) and cortex (Cardin et al. 2009; Sohal et al. 2009). Based on extensive studies in hippocampus, two distinct mechanisms have been identified for the generation of gamma oscillations (Whittington et al. 2000; Bartos et al. 2007; Tiesinga and Sejnowski 2009): a mechanism relying on PV cells only (Interneuron Network Gamma, ING, Wang and Buzsáki 1996; Brunel and Hakim 1999)) and a mechanism relying on the interactions between PV cells and pyramidal cells (Pyramidal-Interneuron Network Gamma, PING, (Tiesinga and Sejnowski 2009)). These mechanisms can co-exist in models of cortical columns (Bos et al. 2016). However, it remains unknown how these mechanisms are embedded in a circuit with multiple interneuron types.

It is likely that the functional interactions between PV, SOM and other cell types are affected by changes in synchrony within the circuit. For example, synchronization of inhibitory cells in the gamma frequency range has been shown to affect gain of model pyramidal cells (Tiesinga et al. 2004). It has recently also been suggested that PV and SOM cells respond differently to synchrony- and rate-coded information; PV cells were most likely to represent information that was coded by synchronized pre-synaptic activity (Tiesinga et al. 2002), while SOM cells responded to both synchrony- and rate-coded information, albeit slower than the PV cells (Tran et al. 2019). The behavior of PV and SOM cells therefore not only depends on the strength of excitatory and inhibitory inputs, but also on the state of the surrounding network. Such mesoscale interactions might, in part, explain why studies into the functional separation between PV and SOM cells have so far produced complex and sometimes contradicting findings. For example, optogenetic studies into the role of PV and SOM cells in gain modulation have yielded confusing results: activation of PV cells led to divisive inhibition in mouse V1, preserving stimulus selectivity in some (Atallah et al. 2012; Wilson et al. 2012), but not other studies (Lee et al. 2012). Similarly, activation of SOM cells could either increase selectivity (Wilson et al. 2012) or leave it unaffected (Lee et al. 2012). It has since been suggested that such discrepancies can stem from differences in how these cell types are recruited by different stimulation protocols, with outcomes depending on the strength (Atallah et al. 2014; Lee et al. 2014b; El-Boustani and Sur 2014) and duration (Lee et al. 2014b; Li et al. 2014). Furthermore, stimulation and suppression of PV and SOM cell activity led to different conclusions about their function in mouse auditory cortex (Phillips and Hasenstaub 2016) and stimulation of PV cells in somatosensory cortex could, in some conditions, lead to an unexpected increase in activity in neighboring pyramidal cells (Mahrach et al. 2020). These studies suggest that interpretation of optogenetic stimulation requires an understanding of interactions between cell types at the network level and how this affects their input–output mapping.

Despite the substantial increase in the amount of experimental data, there is still no comprehensive understanding of how interactions between PV, SOM and pyramidal cells affect synchronization of neural activity at frequencies other than the gamma band. It is also unclear how synchronization is affected by input conditions and connection profiles. Obtaining such understanding is complicated by the fact that (1) interactions cannot be assessed on a pair-by-pair basis, as the state of the motif as a whole can affect the outcome of the pair’s interaction; and (2) the number of possible interactions scales quadratically with the number of cells types. This makes an experimental assessment of interactions extremely complex and time consuming. Suggestions have been made on how to improve assessment and reporting of interactions through the use of circuit motifs (Womelsdorf et al. 2014b; Braganza and Beck 2018).

Here, we use a modeling approach to identify the possible asynchronous and oscillatory outcomes of interactions in circuit motifs of three-cell types: pyramidal cells, and PV and SOM interneurons. We report a wide range of possible circuit behaviors, from robust beta oscillations, to theta–gamma phase-amplitude coupled oscillations, to switches between distinct frequency bands. We document both frequency and amplitude of the oscillations in the LFP, as well as the firing rates and phase-of-firing of the different cell types.

## Methods

### Overview

**Fig. 1:**
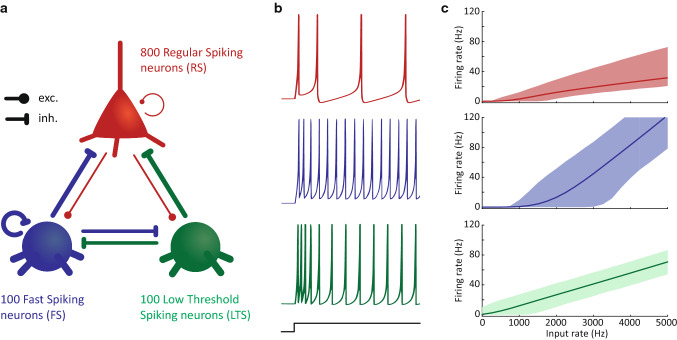
Model neurons and circuit. **a**: Schematic of the full network; **b**: Example traces for the three model cell types in response to a step current; **c**: Firing rates per cell types in response to Poisson input spike trains of between 0 and 5000 Hz (*x* axes). Solid lines give the mean firing rate across all neurons of a cell type (for the number of cells included see panel a) and 10 different random seeds. Shaded areas give the 95% confidence interval. Throughout the manuscript, red represents the regular spiking neurons (RS), blue the fast-spiking neurons (FS) and we use green for the low-threshold spiking neurons (LTS)

To study the network dynamics of two-interneuron neural circuits, we simulated the spiking activity and local field potentials for circuits of 1000 cells. These cells were modeled as Izhikevich point neurons (Izhikevich 2003). Circuits with three-cell types (Fig. 1a) contained 80% model excitatory cells (DeFelipe and Fariñast 1992; Markram et al. 2004), i.e., 800 regular spiking cells, and 20% interneurons, consisting of 100 low-threshold spiking cells (SOM cells) and 100 fast-spiking cells (PV cells). Example voltage traces and f-I curves are given for each of these cell types in Fig. 1b and c. Two-cell-type circuits instead contained 200 cells of one of the two-interneuron types. Neurons were connected via excitatory (AMPA) and inhibitory (GABA) synapses.

**Fig. 2:**
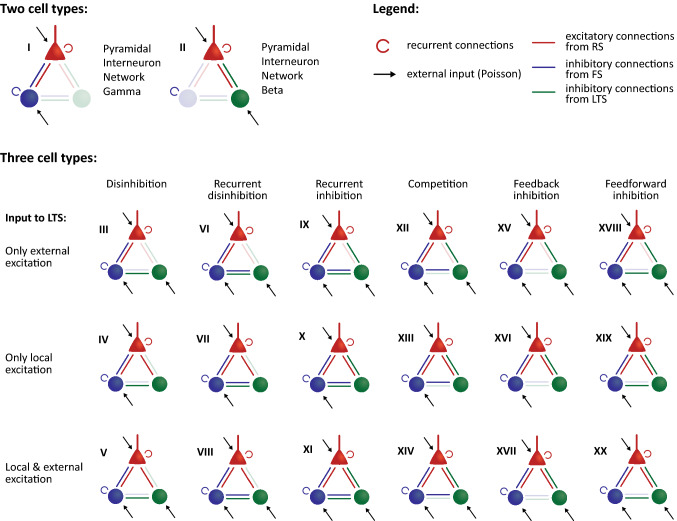
Overview of the circuit motifs and the roman numerals used to identify them throughout this manuscript. The two-cell-type motifs, RS-FS and RS-LTS, are shown on the top row. The three-cell-type motifs are given below and are structured in 3 rows representing (from top to bottom) motifs with external input to LTS cells only; motifs with local excitation to LTS cells only; and motifs with both local and external inputs to LTS cells. The motifs are also structured into columns representing different types of inhibition provided by the LTS cells (discussed in the main text)

We tested the impact of two elements on the dynamics of the two-interneuron circuit, namely the absence or presence of specific connections within the circuit and the presence and strength of external inputs to each of the cell types. Each circuit motif was simulated for a range of inputs to the RS and FS cells, once with external inputs to LTS cells and once without this input. This resulted in a total of 18 three-cell-type motifs and 2 two-cell-type motifs (see Fig. 2).

**Fig. 3:**
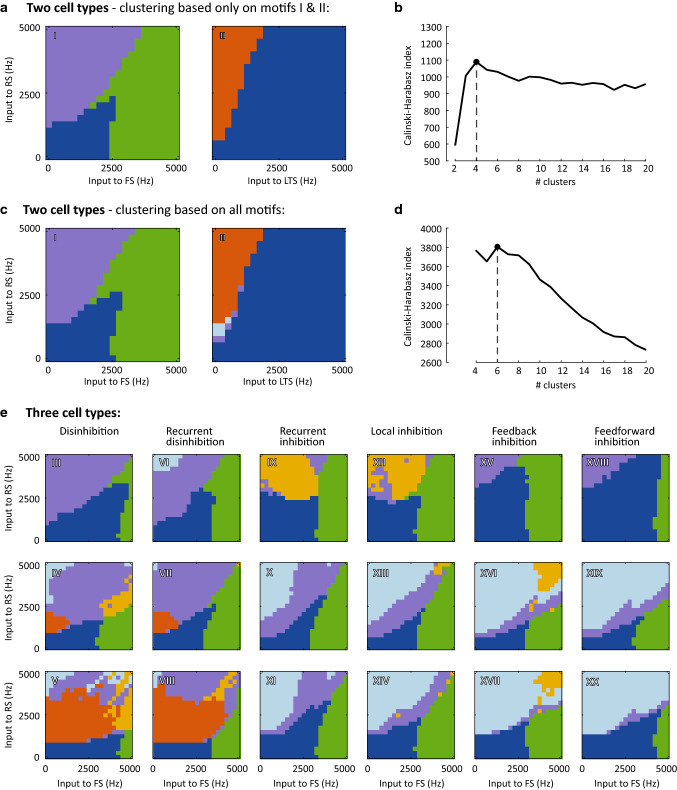
Outcome of *k*-means clustering. **a & b:** *k*-means clustering performed on the two-cell-type motifs only (motifs I and II), using *k* = 1–20. The Calinski–Harabasz index identified *k*= 4 as optimal number of clusters (**b**, dashed line indicates the peak). In **a**, the cluster to which each of input conditions (*x*- and *y*-axes) of motifs I and II was assigned is shown. Clusters are color-coded; **c-e:** *k*-means clustering was subsequently performed on all 20 circuit motifs combined, for *k* = 4–20. The optimal cluster count was here identified at *k*  = 6 (Calinski–Harabasz index, dashed line in **d**). The distribution of these 6 clusters across input conditions of motifs I and II is given in **c** (using a color code matched to a), and the three-cell-type motifs are given in **e**, following the layout from Fig. 2. The cluster color code used here is maintained throughout the manuscript

### Model neurons

The three cell types were modeled using the Izhikevich neuron model and followed the definitions used in (Izhikevich 2003). For a detailed discussion of the characteristics of these neuron models, as well as specific parameter choices for each cell type, we refer to (Izhikevich 2007).

The neurons were modeled through a system of two coupled differential equations:

1 $$ \begin{gathered} \frac{dV}{{dt}} = 0.04V^{2} + 5V + 140 - U + I \hfill \\ \frac{dU}{{dt}} = a\left( {bV - U} \right) \hfill \\ I = \mathop \sum \limits_{j} I_{{{\text{syn}}}}^{j} + I_{{{\text{bg}}}} + I_{{{\text{noise}}}} \hfill \\ {\text{if }}V \ge 30\,{\text{mV, then }}\left\{ { \begin{array}{*{20}c} {V \leftarrow c } \\ {U \leftarrow U + d} \\ \end{array} } \right. \hfill \\ \end{gathered} $$

Here, $V$ is the cell’s membrane potential, whereas $U$ captures slower subthreshold dynamics, and $a,b,c,d$ are parameters. $I$ represents the inputs into the cell, which here consists of the sum of synaptic inputs from other neurons $j$ in the circuit ($I_{{{\text{syn}}}}^{j}$), background synaptic input ($I_{{{\text{bg}}}}$) and noise ($I_{{{\text{noise}}}}$), which are described in more detail in the next two sections. Variables and parameters are dimensionless, but $V$ represents the membrane potential in millivolt, with time $t$ in milliseconds. The $V$-equation models the rising phase of action potentials, but not the falling phase. Instead, a condition is added such that when $V$ exceeds the spike threshold of 30 mV, its value is reset to $c$, while $d$ is added to the value of $U$ to maintain a refractory period.

Initial conditions $V_{0}$ were drawn, for each of the cell types, from a uniform distribution spanning between −80 and −70 mV. The corresponding initial conditions for $U$ were computed as $U_{0} = bV_{0} + d$.

The values of parameters $a,b,c,d$ differed for the three cell types and are given in Table 1. To introduce heterogeneity into the population, some parameters were drawn from uniform or squared uniform distributions, as detailed in Table 1. The model neurons resulting from Eq. 1 and Table 1 have relatively short membrane time constants (in the order of a few millisecond), while reported membrane time constants from neural recordings vary widely, up to several dozen milliseconds, and are often found to be shorter for FS cells than for other cell types (Povysheva et al. 2006; Neske et al. 2015). The impact of the membrane time constant is explored in Fig. 11 (Appendix III).

**Table 1:** Parameter values for the three cell types

Cell type	Parameter			
	a	b	c	d
Regular spiking	0.020	0.20	-65 – 50 *	2 – 8 *^1^
Fast Spiking	0.10—0.18	0.15—0.20	-65	2
Low-Threshold Spiking	0.020—0.025	0.20—0.25	-65	2

Where a range is given, a uniform distribution scaled to the stated range was used, unless the range is followed by a *, in which case a beta distribution was used with shape parameters 0.5 and 1; or *^1^ in which case a beta distribution with parameters 1 and 0.5 was used

### Synapses and connection probabilities

Cells communicated with each other through AMPA and GABA synapses. Synaptic currents $I_{{{\text{syn}}}}$ between pre-synaptic cell $j$ to post-synaptic cell $i$ were modeled as follows:

2 $$ I_{{{\text{syn}}}}^{i,j} \left( t \right) = {\varvec{C}}_{{{\varvec{i}},{\varvec{j}}}} \user2{ }H\left( {t - t_{{{\text{delay}}}} } \right)e^{{ - \frac{{t - t_{{{\text{delay}}}} }}{{\tau_{{{\text{pre}}}} }}}} w_{j} *S^{j} \left( t \right) $$

On the RHS, $S^{j} \left( t \right)$ is the spike train of pre-synaptic neuron $j$, with $S^{j} = 1$ at $t_{spike}$ and 0 otherwise, which is convolved with a synaptic kernel. In this kernel, $H$ is the Heaviside step function, with $t_{{{\text{delay}}}}$ the synaptic delay time constant, which was set to 1 ms for all synapses. The third factor is an exponential decay with characteristic time scale $\tau_{{{\text{pre}}}}$, determined by the pre-synaptic cell type. $\tau_{{{\text{pre}}}}$ was 2 ms for excitatory connections (i.e., from RS cells), 3 ms for inhibitory synapses from FS cells and 6 ms for inhibitory synapses from LTS cells (see Table 2b). The resulting differences in synaptic potential durations between the synapse types quantitatively mimics empirical findings from rodent somatosensory (Silberberg and Markram 2007) and hippocampal (Savanthrapadian et al. 2014) areas and follows previous modeling work (Vierling-Claassen et al. 2010). In Eq. (2), $w_{{\text{j}}}$ represents the synaptic weight, based on the pre-synaptic cell $j$, which was drawn from a normal distribution, with averages and standard deviations given in Table 2b. The post-synaptic potentials resulting from single pre-synaptic action potentials are characterized in Fig. 9a and b in Appendix I. ${\varvec{C}}_{{{\varvec{i}},{\varvec{j}}}}$ represents the connection matrix. The connection matrix was generated randomly, i.e., without any spatial structure, following the probabilities given in Table 2a, which were based on (Pfeffer et al. 2013). Depending on the circuit motif, specific connections between cell types were left out or introduced (see Fig. 2 for all circuit motifs), we did, however, not vary the connection probabilities, i.e., whenever a connection existed, it followed the probability stated in Table 2a.

**Table 2:** Connection probabilities (**a**) and synaptic current parameters (**b**) for each of the cell types

a		Pre			b	Pre-synaptic cell type	${\varvec{w}}_{{{\text{pre}}}}$(avg ± SD)	${\varvec{\tau}}_{{{\text{pre}}}}$(ms)
	Post	RS	FS	LTS				
	RS	5%	30%	40%		RS	1 ± 0.5	2
	FS	10%	30%	20%		FS	−2 ± 1	3
	LTS	10%	20%	0%		LTS	−2 ± 1	6

### External inputs

In addition to local synaptic inputs, cells also received two ‘external’ input currents, $I_{{{\text{bg}}}}$ and $I_{{{\text{noise}}}}$. The former, $I_{{{\text{bg}}}}$, represents driving synaptic inputs from excitatory cells from other regions of the brain, for example input from the thalamus. It was modeled as Poisson spike trains arriving via AMPA synapses, using Eq. (2) with $C$ = 1, $w$ = 1 and $\tau_{{{\text{pre}}}}$ = 2 ms. Each cell received an independently generated Poisson spike train. The firing rate of the spike train was varied and ranged between 0 and 5000 Hz, in 250 Hz steps. This input rate mimics the combined inputs from all synaptic inputs a cell might receive, which generally are thought to run in the thousands (DeFelipe and Fariñast 1992).

A noise current was inserted in all neurons to mimic all other sources of variability in neural responses. This noise source consisted of two parts: $I_{{{\text{noise}}}} = I_{{{\text{offset}}}} + I_{{\text{t}}} \left( t \right)$. Here, $I_{{{\text{offset}}}}$ is a static offset per neuron, drawn from a normal distribution and $I_{{\text{t}}} \left( t \right)$ is a time-varying noise source drawn independently at every time step from a normal distribution with mean 0 and a standard deviation of 1 mV. The variations in membrane potential caused by the noise current are characterized in Fig. 9c and d in Appendix I. The noise source $I_{{{\text{noise}}}}$, as well as the variability in neuron parameters $a - d$ and synaptic weight parameter $w_{{\text{j}}}$, introduce variability in the membrane potential at the time scale of the membrane time constant and therefore can affect the timing of individual spikes. This variability was added to ensure that identified network states were generalizable and did not rely on individual input- or connection patterns.

### Model output: spike times and local field potentials

Spikes were detected whenever the voltage $V$ of a model neuron crossed the 30 mV threshold (see Eq. (1)). Time stamps and neuron IDs of all spikes were stored for further analysis. In addition to spike times, we also stored the mean voltage trace across all cells, which was used as a proxy for the Local Field Potential (LFP), as would be recorded using an invasive extracellular recording setup. We chose to use the mean voltage here, because this measure was cheap to compute, allowing for the high number of simulations used here, and was shown to perform similarly to the more expensive weighted synaptic current under the high spiking condition generated by the model network (Mazzoni et al. 2015).

### Simulations

The above differential equations were numerically integrated using the Euler method with a time step of 0.2 ms. Simulated time series were 2300 ms long, of which the first 300 ms were discarded before further analysis. For every circuit design, we simulated time series for a range of external input values. Simulations for different input values were run using the same random seeds, to improve comparison between input values. Every simulation was repeated 10 times with different random seeds (i.e., with different connection patterns and noise currents). Unless stated otherwise, results shown represent the average across these 10 repetitions.

### Analyses—LFPs

We analyzed the spectral content of the LFP for each simulation. After exclusion of the first 300 ms of the simulation, we computed the power spectral density using a multitaper approach with a time-halfbandwidth (NW) product of 3. We then identified the frequency with highest power in the full spectrum, as well as in two pre-defined frequency bands: a low-frequency band between 2 and 30 Hz and a high-frequency band between 30 and 150 Hz. The three peak frequencies and the corresponding power were stored.

For simulations that had sufficient power (at least 1 dB/Hz) for both the low and high peak frequency, and for which the peak frequency in the higher frequency band was above 40 Hz and not the first harmonic of the lower peak frequency, we subsequently computed phase-amplitude coupling (PAC). We used the weighted Phase Locking Factor (wPLF; Maris et al., 2011) between the phase of the low-frequency band and the amplitude from the high-frequency band as a measure of PAC. Briefly, (1) we computed the instantaneous phase in the low-frequency band by zero-phase filtering the LFP with a second-order Butterworth filter with a pass band of 2–30 Hz, subtracting the mean, computing the Hilbert transform of the filtered signal and normalizing the Hilbert transformed signal by dividing by its norm; (2) we computed the instantaneous amplitude for the high-frequency band by zero-phase filtering the LFP with a fourth-order Butterworth filter with a pass band of 30–150 Hz, subtracting the mean, computing the Hilbert transform and taking the absolute value divided by the norm of the Hilbert transformed signal; (3) we computed the PLF by taking the absolute value of the inner product between the signal obtained in steps 1 and 2.

### Analyses—spikes

To characterize the spiking behavior of the 3 cell types in each of the motifs, we report the firing rate, as well as the burst fraction per cell type. The firing rate was computed as the average number of spikes per second across all cells in the circuit belonging to a given type. The burst fraction was determined by identifying all spikes of a single neuron that occurred within 10 ms of each other and by dividing the resulting number of burst events by the sum of the number of bursts and the number of single spikes. This resulted in a number between 0, when all spikes were single spikes, and 1, when all spikes occurred in bursts. The computation of burst fraction required each neuron to have at least 2 spikes to be detectable.

In addition, we computed two spike-LFP measures for every simulation: the pairwise phase consistency (PPC; Vinck et al. 2010), which gives an indication of how strongly the individual cell types were locked to the LFP; and the average phase of firing. Both PPC and average phase of firing were computed using the instantaneous phase at the peak frequency of the LFP. To obtain the instantaneous phase, we zero-phase filtered the LFP trace with a second-order Butterworth filter with a 10 Hz wide passband centered around the peak frequency. After mean-correction, we computed the Hilbert transform and took the angle to obtain the instantaneous phase. We then identified the phases $\theta_{t}^{c}$ at all spike timestamps $t$ from each cell type $c$. Note that a phase of 0 means that the cell spiked at the positive peak of the LFP, and a phase of $\pm \pi$ resulted from spiking around the trough of the oscillation in the LFP. To obtain the average phase of firing, we computed the circular average: $\theta_{{{\text{avg}}}}^{c} = {\text{Arg}}\left( {\mathop \sum \nolimits_{t} e^{{i\theta_{t}^{c} }} } \right).$

The PPC was then computed, following (Vinck et al. 2010): ${\text{PPC}}^{c} = \frac{1}{{N_{s}^{c} }}\mathop \sum \nolimits_{i,j} {\text{cos}}(\theta_{i}^{c} - \theta_{j}^{c}$), where $N_{s}^{c}$ is the total number of spikes from cell type $c$ and $i,j$ denote a pair of spike time stamps.

### Clustering

To compare the many different input regimes and connection profiles (see Fig. 2) with each other efficiently, we opted for a clustering approach. We used *k*-means clustering with the following characteristics, obtained from the spikes and LFP produced by the model, as features:

- Firing rates for each of the three-cell types
- PPC for each of the cell types
- Burst fraction of individual cells for each of the cell types
- Peak frequencies in the low (2–30 Hz) and high (30–150 Hz) frequency bands
- Log-transformed power at the peak frequencies in the low- and high-frequency bands
- Phase-amplitude coupling between the low- and high-frequency bands

Each of these measures was first averaged across the 10 different seeds and subsequently standardized, to provide equal weighing across the features. When measures could not be calculated in at least 5 of the seeds, for example because a cell type was inactive, values were set to 0. We then concatenated all input conditions from all connection profiles and entered this into the *k*-means clustering algorithm using a squared Euclidean distance measure. We ran the *k*-means algorithm 10 times, to account for initial conditions of the clusters, with a maximum of 1000 iterations.

To determine the appropriate number of clusters, we first clustered only the two two-cell-type motifs (Fig. 2a). We repeated the clustering on these two motifs for 1 to 20 clusters and evaluated the appropriate number of clusters using the Calinski–Harabasz index (Caliński and Harabasz 1974). This resulted in an optimum of 4 clusters (Fig. 3b), with 2 clusters unique to motif I, 1 cluster unique to motif II and 1 cluster appearing in both motifs (Fig. 3a). We then clustered the entire dataset (i.e., using all motifs) using 4 to 20 clusters and established, again using the Calinski–Harabasz index, that the optimal number of clusters was 6.

### Software

All simulations were run using MATLAB 2018a (The Mathworks) with the Signal Processing Toolbox, using standard functions and custom code. For the plots in Fig. 5 and S1, the violin.m function by H. Hoffmann (Hoffmann 2015) was used. All custom code is available via https://github.com/marijeterwal/RS-FS-LTS-clustering (https://doi.org/10.6084/m9.figshare.14695584).

## Results

### Dynamics of 20 unique circuit motifs were modeled using spiking neuron models

We aimed to characterize the oscillatory behavior and corresponding circuit dynamics of all generic circuit motifs consisting of pyramidal cells, as well as PV and SOM inhibitory neuron types. To this end, we identified all possible circuit motifs containing these three cell types and simulated their activity patterns. We compared the activity produced by the three-cell-type model to simulations of the well-studied motif consisting of pyramidal cells and inhibitory PV basket cells (Fig. 2, motif I).

To arrive at a comprehensive overview of the dynamics that can be achieved by the three-cell type circuit, we did not make assumptions about the connection pattern between the SOM cells and the two other cell types. Instead, we included all possible connection patterns separately and simulated the activity for all of them. Nor did we assume local excitation or long-range inputs (i.e., from outside the local circuit) to the SOM cells; again, we simulated both possible input conditions as separate motifs. This resulted in a total of 18 unique three-cell-type motifs, shown in Fig. 2, with columns representing the different connection patterns between the cell populations and rows showing the different input regimes to the SOM cells.

We modeled each of the motifs using randomly connected Izhikevich point neuron models (Izhikevich 2003). We used regular spiking (RS, Fig. 1, red) excitatory cells to model pyramidal cells, fast-spiking cells (FS, Fig. 1, blue) to match PV cells and low-threshold spiking cells (LTS, Fig. 1, green) to represent SOM cells. We simulated the spiking activity and local field potentials (LFPs) of the circuits for 2 s periods across a range of different inputs to the RS and FS cell populations and repeated this 10 times with different random seeds. We analyzed the spiking activity and the modeled LFPs of each of the three-cell-type motifs at each input condition and compared them with the well-known two-cell-type motif consisting of RS and FS cells, as well as to a circuit consisting of RS and LTS cells.

### K-means clustering identifies known types of circuit dynamics in RS-FS motifs

In order to compare and qualitatively describe the behaviors generated by all 20 circuit motifs, we opted to use a *k*-means clustering approach. This unsupervised clustering approach allowed us to process the large amount of data produced by the 20 circuit motifs and identify patterns of activity within and across different motifs. As a result, we could group the behaviors of all circuit motifs into a small number of characteristic activity patterns in a data-driven way.

Briefly, the clustering approach involved the following steps: Firstly, we identified key features of the motif’s activity, such as firing rate and oscillation frequency, for each input condition and motif. We then concatenated these features from all input conditions and motifs to create one large dataset. We entered this combined dataset into a *k*-means clustering algorithm. As features, we used both spike-based and LFP-based measures. The spike-based measures were firing rate, pairwise-phase consistency (PPC, this quantifies the locking of a cell type to the LFP’s oscillations) and single-cell burst fraction, for each of the cell types separately. As network activity characteristics based on the LFP, we used peak power and the corresponding oscillation frequency within the lower (2–30 Hz) and higher (30–150 Hz) frequency bands, and phase-amplitude coupling between those bands (see Methods for details). The values for each of those measures were averaged across the 10 repeated simulations (with different random seeds) and were only included for clustering when they could be determined in at least 5 of the simulations to avoid bias toward outliers.

To validate the clustering method, we first clustered only the two-cell-type motifs, I and II, as the network dynamics of these motifs are well-established and distinctive. The dynamics of the RS-FS motif are particularly well-characterized. This circuit motif has been shown to robustly synchronize in the gamma frequency range through two possible mechanisms (Brunel 2000; Whittington et al. 2000; Bartos et al. 2007; Tiesinga and Sejnowski 2009): Interneuron Network Gamma (ING), which critically depends on recurrent inhibition in the basket cell population (Wang and Buzsáki 1996; Brunel and Hakim 1999); or Pyramidal cell–Interneuron Network Gamma (PING) mechanism, which depends on interactions between the excitatory or inhibitory populations. The motif can also generate asynchronous behavior, and the type of circuit dynamics depends on the amount of input the two-cell types receive.

**Fig. 4:**
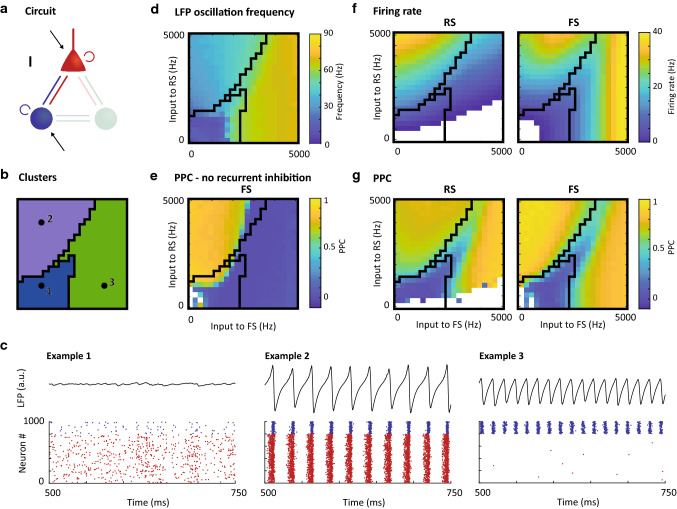
Characteristics of motif I: *K*-means clustering reproduces known types of circuit dynamics in RS-FS motifs. **a**: Schematic of the circuit motif (repeated from Fig. 2); **b**: Clustering result for this motif (repeated from Fig. 3); **c**: Example LFPs (top) and raster plots (bottom) for 3 input settings (indicated by black dots in panel **c**). Red dots are spikes from RS cells, blue dots are FS cells; **d**: Peak frequency per input setting identified based on the LFP; **e**: Pairwise phase consistency of spikes relative to the LFP at peak frequency for fast-spiking cells for a special condition without FS-FS inhibitory connections (compare with g, right). Cluster outlines for the full circuit in a are overlaid for comparison; **f**: Average firing rate for RS cells (left) and FS cells (right). **g**: Pairwise phase consistency of spikes relative to the LFP at peak frequency for regular spiking cells (left) and fast-spiking cells (right); White areas in **e**–**g** did not allow for analysis in a sufficient number of the random seeds (see Methods). Black lines in **b**, **d**–**g** give the outlines of the clusters

**Fig. 5:**
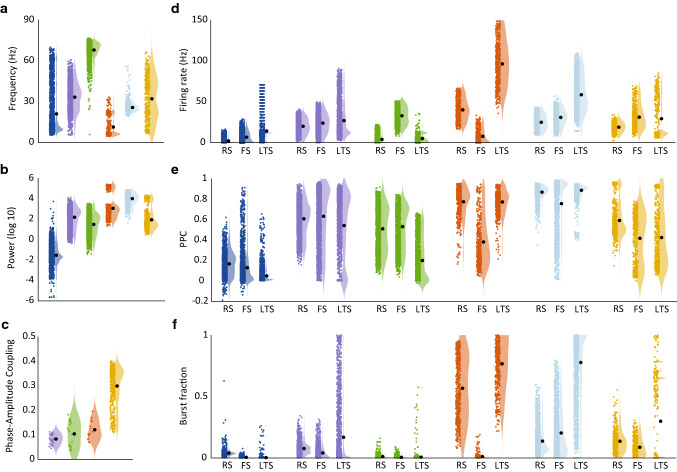
Characteristics of spiking activity, LFP oscillations and spike-LFP measures per cluster. Using the same color code for the clusters as in Fig. 3, the LFP peak frequency (**a**, see also Fig. 10 in Appendix II), corresponding power (**b**), phase-amplitude coupling (**c**) between phase of the lower (2–30 Hz) and amplitude of the higher frequency band (30–150 Hz) are given for each of the clusters, as well as firing rate (**d**), pairwise phase consistency between spikes and LFP at peak frequency (**e**) and burst fraction (**f**) are given for each of the three cell types. In addition to plotting all observations (colored dots), the data are represented as violin plots (shaded areas), as well as by the mean across all observations in a cluster (black dots)

We ran *k*-means clustering on the simulated activity of motifs I and II with *k* = 1–20, i.e., assuming between 1 and 20 clusters of activity patterns. Using the Calinski–Harabasz index, we identified that the behavior of the two two-cell type circuits was best described by 4 activity clusters (Fig. 3b). The input conditions we simulated for Motif I (the RS-FS motif) were split up into 3 clusters of approximately equal size (Fig. 3a, left, each color is one cluster). Figure 4 shows the dynamics of Motif I in more detail, with examples of the LFP and raster plots for each of the three clusters given in Fig. 4c. Indeed, the three clusters of this circuit motif showed distinct behaviors (compare the cluster outlines from Fig. 4b to the data in Fig. 3d–g):

- Dark blue cluster: This activity cluster was characterized by low firing rate in both RS and FS cell populations (Fig. 4f) and low synchronization among the cells, as indicated by the low PPC for both RS and FS cells (Fig. 4g) and the low power for the blue cluster in general (Fig. 5b), matching an asynchronous state;
- Purple cluster: With both RS and FS cells active in approximately equal amounts (Fig. 4f), this cluster showed strong synchronization in the gamma frequency range (Fig. 4d), with both cells locking to this rhythm (Fig. 4g). The synchronization at the input conditions in this cluster survived when the recurrent connections between FS cells were broken (Fig. 4e). These characteristics suggest this cluster represents the PING mechanism;
-  Green cluster: like the purple cluster, for input conditions in the green cluster the circuit showed synchronized activity in the gamma frequency range across all input conditions, but spiking of RS cells was infrequent or absent in this cluster. FS cells did show high firing rates and high PPC. The synchronization in this cluster was broken by removing recurrent FS connection from the circuit (Fig. 4e), demonstrating that this cluster represents the ING mechanism.

**Fig. 6:**
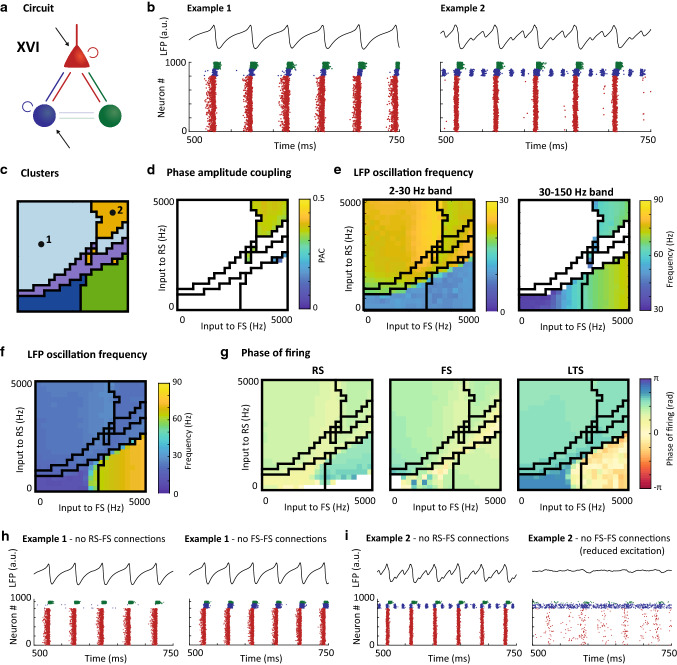
Characteristics of motif XVI: local excitation of LTS cells generates stable beta oscillations and beta-nested gamma oscillations. **a**: Schematic of the circuit motif (repeated from Fig. 2); **b**: Example LFPs (top) and raster plots (bottom) for 2 input settings (indicated by black dots in panel c). Red dots are spikes from RS cells, blue dots are FS cells and green dots are spikes from LTS cells; **c**: Clustering result for this motif (repeated from Fig. 3); **d**: Phase-amplitude coupling (PAC) between phase of low frequency and amplitude of high-frequency band (note that the color code is truncated at 0.5); **e**: Peak LFP frequency identified after filtering in the low-frequency band (2–30 Hz, left) and the high-frequency band (30–150 Hz, right). Note that the color bars differ for the left and right panel; **f:** Peak LFP frequency without frequency restrictions; **g**: Mean phase of firing relative to the phase of the LFP at peak frequency for regular spiking (RS, left), fast-spiking (FS, middle) and low-threshold spiking cells (LTS, right). White areas in d, e and g did not allow for analysis for a sufficient fraction of the random seeds (see Methods). Black lines in c-g give the outlines of the clusters; **h:** LFP (top) and raster plot (bottom) of example 1 without RS-to-FS connections (left) and without FS-to-FS connections (right), illustrating the beta oscillation in example 1 is not dependent on interactions between RS and FS; **i:** as h, but for example 2, illustrating the beta-nested gamma oscillations rely on FS-to-FS connections, i.e., an ING mechanism. In the right panel, excitation to both RS and FS populations was reduced to 2500 Hz to compensate for the increased inhibition in the circuit

**Fig. 7:**
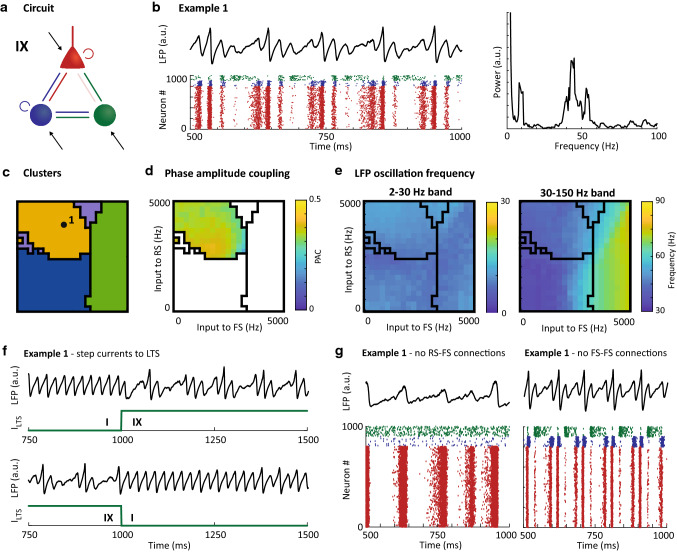
Characteristics of motif IX: Feedforward inhibition of pyramidal cells with FS to LTS inhibition generates theta-nested gamma oscillations. **a**: Schematic of the circuit motif (repeated from Fig. 2); **b**: Example LFP (top), raster plot (bottom) and the frequency spectrum (right) for the input setting indicated by the black dots in panel c. Red dots are spikes from RS cells, blue dots are FS cells and green dots are spikes from LTS cells; **c**: Clustering result for this motif (repeated from Fig. 3); **d**: Phase-amplitude coupling (PAC) between phase of low frequency and amplitude of high-frequency band (note that the color code is truncated at 0.5). The white area did not allow for analysis in a sufficient number of the random seeds (see Methods); **e**: Peak frequency per input setting identified based on the LFP in the low-frequency band (2–30 Hz, left) and the high-frequency band (30–150 Hz, right). Note that the color bars differ for the left and right panel. Black lines in c-e give the outlines of the clusters; **f:** Examples of external step currents applied to the LTS population, with RS and FS inputs as in b. The LFP is shown on the top row, the external current to LTS below. Without external current, the circuit behaves like PING in motif I (compare with Fig. 4), while with external current, the theta–gamma oscillation appears (compare with panel b); **g:** LFP (top) and raster plot (bottom) of input conditions from example 1 without RS-to-FS connections (left) and without FS-to-FS connections (right), illustrating the theta-nested gamma oscillations in example 1 is dependent on RS-FS, but not FS-FS interactions, suggesting a PING mechanism

**Fig. 8:**
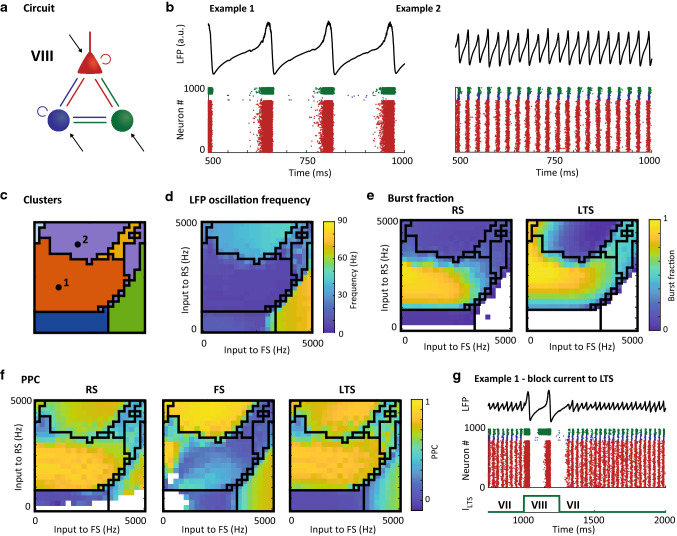
Characteristics of motif VIII: Disinhibition via LTS cells induces bursting in RS and LTS cells. **a**: Schematic of the circuit motif; **b**: Example LFPs (top) and raster plots (bottom) for 2 input settings (indicated by black dots in panel c). Red dots are spikes from RS cells, blue dots are FS cells and green dots are spikes from LTS cells; **c**: Clustering result for this motif (repeated from Fig. 3); **d**: Peak frequency per input setting identified based on the LFP; **e**: Burst fraction (fraction of total number of bursts and single spikes) for regular spiking cells (left) and fast spiking cells (right); **f:** Pairwise phase consistency of spikes relative to the LFP at peak frequency for regular spiking (left), fast-spiking (middle) and low-threshold spiking cells (right). White areas in e–f did not allow for analysis in a sufficient number of the random seeds (see Methods). Black lines in c-f give the outlines of the clusters; **g:** Example LFP (top row) and raster plot (middle row) of the behavior of motif VIII with and without external input to LTS (bottom row). A short block current switches the circuit temporarily from the PING mechanism displayed by motif VII (which has no external current to LTS) to the bursting behavior of motif VIII

These results show that (1) the circuit model containing simple RS and FS neuron models reproduced both the asynchronous network activity, as well as synchronous activity through the ING and PING mechanisms identified by previous studies; and (2) that these three categories of activity were correctly identified by the *k*-means clustering approach.

The second two-cell-type motif, containing only RS and LTS cells, is not expected to be physiologically relevant, but we included it in the validation step to allow us to check that the clustering approach correctly separated synchronization driven by FS cells from synchronization driven by LTS cells. Indeed, *k*-means clustering identified a unique cluster (orange, Fig. 3a right) for input conditions of motif II where RS and LTS cells were both active (see Fig. 5d) and producing an oscillation (see Fig. 5b for LFP power and Fig. 5e for PPC). The orange cluster will be discussed in more detail later. Note that LTS has no recurrent connectivity in motif II, and hence cannot synchronize through an ‘ING-like’ mechanism. High LTS input conditions are therefore led to high, but asynchronous, firing in the LTS cells, with little or no firing in the RS cells. *K*-means clustering correctly grouped these asynchronous input conditions of motif II together with the asynchronous dark blue cluster found for motif I.

In addition to validating our model and clustering method, the clustering results from motif I and II also provided a reference against which we compared the clustering results obtained from all 20 motifs combined, which are shown in Fig. 3c and e. In this second clustering step, we clustered all input conditions of 20 motifs together using *k* = 4–20 clusters. The Calinski–Harabasz index indicated that 6 was the optimal number of activity clusters when combining the data from all motifs. As shown in Fig. 3c, the input conditions of motif I were clustered in virtually identical ways when clustering only data from motifs I and II (shown in Fig. 3a), or when using data from all motifs (Fig. 3c). The clustering results also showed a very high overlap for the orange and dark blue RS-LTS clusters.

Our validation steps show that *k*-means clustering based on a small set of spike-based and LFP-based activity characteristics can correctly identify (1) known oscillatory mechanisms in the well-studied RS-FS motif; and (2) separate activity patterns driven by RS-FS interactions from activity patterns driven by RS-LTS interactions. Importantly, it also identifies these mechanisms correctly when including the data from all three-cell-type motifs, suggesting the *k*-means clustering is a robust approach to identifying activity patterns in the three-cell-type motifs.

### Adding SOM cells to the circuit produced three new synchronization patterns

The *k*-means clustering approach identified, in addition to the 3 well-known activity patterns from the RS-FS circuit, 3 additional clusters, indicated by orange, light blue and yellow in Fig. 3. Of these new activity patterns, the orange cluster was already seen for the RS-LTS cluster and is hence expected to rely strongly on an interaction between RS and LTS cells. On the other hand, the yellow and light blue activity patterns are unique to the triplet of RS, FS and LTS cells.

To gain insight into the kinds of behaviors captured by these newly identified clusters of circuit behaviors, the characteristics of spiking activity and LFPs are given in Fig. 5 for each of the clusters and across all motifs, using the color code from Fig. 3. Interestingly, with the exception of the dark blue cluster that was already seen in the RS-FS motif and represents asynchronous activity, all other clusters represent activity patterns with high oscillatory power (Fig. 5b) and high PPC (Fig. 5e). This suggests that the new yellow, orange and light blue clusters represent distinct patterns of synchronized activity.

These five clusters representing synchronous activity covered a wide range of different oscillation frequencies (Fig. 5a). Across clusters, these frequencies were not uniformly distributed, but were concentrated in the frequency bands observed in electrophysiological recordings, particularly in the theta/alpha band, high beta band and high gamma band (see Fig. 10 in Appendix II). As seen before, the well-known PING cluster (purple) covered high beta and low gamma frequencies, while the green ING cluster contained both low and high gamma frequencies. On the other hand, two of the new clusters were dominated by the lower frequencies: the orange cluster covered theta (in the three-cell-type circuits) to beta (in motif II) frequencies and the light blue cluster almost exclusively covered beta frequencies. The yellow cluster showed a bimodal distribution of oscillation frequencies, with a group of theta/alpha/beta frequencies and a group of low gamma frequencies (we will discuss this in more detail below). The yellow cluster could instead be distinguished from the purple cluster, which covers a similar frequency range, by a high phase-amplitude coupling (Fig. 5c), which will be discussed in more detail later.

In addition to differences in oscillation frequency and power, the clusters also showed distinct patterns of spiking activity in the different cell types (Fig. 5d). In line with previous studies, the green ING cluster consistently showed high FS cell firing, but contained a high number of observations with no RS and/or LTS firing. The purple PING-cluster, known to depend on RS-FS interactions, showed approximately equal firing rates for the RS and FS populations, which co-occurred with both low and high LTS firing rates. On the other hand, the new light blue and yellow clusters had nonzero firing rates for all three-cell types, in agreement with the fact that these clusters were unique to the three-cell-type motifs. The orange cluster, which was also found in some three-cell-type motifs as well as in the RS-LTS motif, showed no or minimum FS firing across the three-cell-type motifs, setting it apart from the light blue cluster. The orange and light blue cluster both showed a high fraction of bursts fired by the RS and LTS, respectively, LTS cell population.

In conclusion, the *k*-means clustering algorithm identified one cluster of asynchronous activity patterns (dark blue) and 5 synchronous activity patterns, across a wide range of motifs and input conditions. While the purple and green clusters showed high agreement with known PING, respectively, ING gamma oscillations, the yellow, light blue and orange clusters represent three distinct oscillatory patterns at low oscillation frequencies and characterized by differences in firing rate and burst fraction of the different cell types. More detailed descriptions and examples of dynamics within the light blue, yellow and orange clusters will be given in the next sections.

### RS-LTS interaction generates stable beta oscillations

The most prevalent new cluster observed in the three-cell-type motifs was the light blue cluster, appearing in all motifs with RS-to-LTS connections. However, the light blue cluster was virtually absent from the RS-LTS motif, suggesting that it relies on interactions between all three-cell types in the circuit. In line with this, the light blue cluster had high firing rates and PPC for all three-cell types (Fig. 5d and e). As shown in Fig. 5a and Fig. 10 (Appendix II), the light blue cluster was characterized by oscillations in the higher beta frequency.

To illustrate the dynamics of the light blue cluster, Fig. 6 shows oscillatory and spiking behavior of Motif XVI. As illustrated in example 1 in Fig. 6b (left), in this cluster the cell types spiked in a consistent order: the RS population spiked first, followed by the FS at a small delay (see also Fig. 6g). Like the FS population, the LTS population was also recruited by the spiking of the RS cells, but this resulted in a slower and more prolonged period of spiking in these cells, as shown by relatively late phase of firing for LTS across the light blue cluster conditions in this motif (Fig. 6g right). This prolonged inhibitory activity is expected to postpone the recovery of the RS population, leading to longer oscillation periods. This period of extended inhibition caused by LTS activity can explain the slow 20–30 Hz oscillation frequency range for motif XVI (Fig. 6f) and was very stable across all motifs with light blue clusters (see Fig. 5a).

Interestingly, and in line with the observed order of spiking, the beta oscillations persisted when RS-to-FS and FS-to-FS connection were broken (Fig. 6h for LFP and raster of example 1). This suggests that the observed beta oscillations were not reliant on interactions between RS and FS cells, but instead were the result of RS-LTS interactions. The model therefore predicts that the introduction of an RS-LTS loop into the three-cell-type circuit results in highly perturbation-resistant oscillations the beta frequency range.

The behavior grouped in the light blue cluster mostly occurred at input conditions that generated PING oscillations in motif I, i.e., at low FS inputs and medium to high inputs to RS (compare purple cluster in Fig. 3c with light blue clusters in e). In most of the motifs in which this behavior appears, these beta oscillations could only be substantially altered by increased excitation to the FS population, i.e., by moving rightward in the cluster plots in Fig. 3e. Such increased FS excitation will often result in a transition to the green cluster, i.e., in the appearance of ING oscillations (green cluster). Alternatively, a switch to the yellow cluster will occur, which leads to beta-nested ING oscillations (Fig. 6b, right), which will be described in the next section.

### Feedforward inhibition of pyramidal cells combined with FS to LTS inhibition generates theta-nested gamma oscillations

The second oscillatory signature that was unique to the three-cell type circuit, the yellow cluster, occurred in seven of the three-cell-type motifs. The yellow cluster was characterized by spiking activity in all three-cell types (Fig. 5d). It stands out from all other identified oscillatory signatures by displaying high phase-amplitude coupling (PAC), i.e., coupling between the phase of the low 5–30 Hz frequency band and the amplitude of the high 30–150 Hz frequency band (Fig. 5c).

The dynamics of one of the 7 three-cell-type motifs included in the yellow cluster, motif IX, is shown in Fig. 7. The high phase-amplitude coupling is apparent in the example traces shown for this circuit in Fig. 7b, with both the LFP and raster plot showing periods of high-frequency activity nested within a slower oscillation. The spectrum for this example revealed a sharp peak at 8 Hz as well as a peak around 45 Hz, confirming that the high PAC was not caused by broad-band activity, but rather by nesting of two oscillations, namely a theta and a gamma oscillation. The PAC was high for all input conditions included in the yellow cluster for this circuit (Fig. 7d). Both the low and high peak frequencies were mostly stable (Fig. 7e), with the low peak frequencies falling within the theta/alpha range (4–12 Hz), and the high-frequency consistently in the lower gamma range.

The theta–gamma coupling shown in Fig. 7 relied on the presence of LTS cells in the circuit, and specifically on the relatively strong coupling between $V$ and $U$ variables for this cell type (*b* parameter), which generates negative feedback on the membrane potential in a way that is analogous to the h-current in hippocampal O-LM cells (Izhikevich 2003). Reducing the *b* parameter to match the FS cells switched the theta–gamma coupled state to a gamma oscillation (Fig. 13 in Appendix III). In line with previous modeling studies, the theta–gamma coupling could, however, be recovered by using a long synaptic time constant for the LTS projections (Fig. 13 in Appendix III), which have been shown to be physiologically feasible for SOM-expressing Martinotti cells (Silberberg and Markram 2007). In addition, theta–gamma coupling relied on sufficient strength of the projections from LTS cells to other cells, and/or sufficient activity in the LTS population (Fig. 14 in Appendix III).

The three-cell-type motifs that show yellow cluster activity cover several different connectivity patterns (Fig. 3e). On the one hand, in motifs IX and XII, the LTS populations received only external inputs and received local inhibition from the FS population. In these circuit motifs, theta–gamma oscillations occurred for low to medium input strengths to FS cells combined with relatively high input to the RS population. Note that the theta–gamma cluster covered a wide range of input conditions and largely overlapped with the input conditions of the PING mechanism (purple cluster) in motif I. This provides us with testable predictions about the circuit behavior of motifs IX and XII. The basis for these predictions is that, without external excitation to the LTS population, the motifs IX and XII (in fact, all motifs on the first row in Fig. 2b and Fig. 3e) have the same functional connectivity as motif I, namely consisting of interconnected RS and FS populations. Motifs IX and XII can therefore be ‘switched’ to behave as motif I by preventing excitation of the LTS population. This suggests that activity of three-cell-type motifs IX and XII, when demonstrating PING oscillations, can switch to a theta-nested gamma oscillation when the external drive to the LTS population is increased. This is demonstrated in Fig. 7f (left) for one input condition. Conversely, the model predicts that theta–gamma nested activity can be switched to PING oscillations by inhibiting the LTS population until it stops spiking (Fig. 7f right), but this cannot produce ING or beta oscillations, nor can it lead to asynchronous behavior.

In the remaining motifs with yellow cluster dynamics, high PAC occurred at high input conditions for both RS and FS cells, setting them apart from motifs IX and XII. An example was given for motif XVI in Fig. 6b (example 2). Similar to the example in Fig. 7, the LFP in Fig. 6b showed a fast oscillation nested within a slower oscillation, but the slow oscillation had a higher frequency than in for motif IX in Fig. 7; for this motif, the base frequencies fell between 20 and 25 Hz, substantially higher than the theta frequencies shown earlier. As evident from the raster in Fig. 6b, the fast oscillation also differed from the behavior in motif IX, by only involving the FS cell population. Indeed, breaking the RS-to-FS connections did not affect the nested oscillation (shown for example 2 in Fig. 6i, left), while removing the FS-to-FS connections resulted in loss of the gamma oscillations (Fig. 6i right), confirming the presence of an ING mechanism nested within the beta-oscillation.

In summary, the yellow cluster was characterized by oscillation frequencies in the low theta/alpha and beta frequency range, and critically, by high phase-amplitude coupling between the phase of the low and amplitude of the high-frequency bands. The spiking patterns suggest that the yellow cluster incorporates both PING and ING gamma oscillations nested within slow oscillations: PING oscillations nested within theta/alpha oscillations in motifs IX and XII, relying on FS-LTS connection, and ING oscillations nested within beta oscillations in the other motifs with yellow clusters, relying on local drive of the LTS cells.

### Disinhibition via LTS cells induces bursting in RS and LTS cells

The third newly identified circuit dynamics is grouped into the orange cluster. This cluster is characterized by the absence of FS firing (Fig. 5d), with both RS and LTS populations active, creating a slow oscillation. This pattern hence dominated motif II, but also appeared in 4 of the 18 three-cell-type motifs (Fig. 3e), although the oscillation frequencies were lower than in motif II (theta and beta range, respectively). In these motifs, LTS cells received excitation from the RS population, and in turn inhibited the FS population, but not the RS cells.

As an example of the behavior of the three-cell-type motifs with RS-LTS oscillation, Fig. 8 shows the circuit dynamics of motif VIII across all input conditions. For this motif, the RS-LTS oscillation covered a wide range of input conditions (Fig. 8b). Across the entire cluster, oscillations showed a stable theta frequency (5–8 Hz, Fig. 8d), with sharp transitions to PING gamma frequencies for increased drive to the RS population (*y*-axis) and to ING gamma frequencies for increased drive to the FS population. The sharp transitions between the activity patterns were also visible in the pairwise phase consistency of the individual cell types (Fig. 8f), with low PPC for the FS cells within the orange cluster. Furthermore, the orange cluster was characterized by high bursting of both RS and LTS cells (Fig. 8e); on average, every cell fired a burst of spikes around the peak of the cycle, followed by period of inhibition (Fig. 8b, example 1).

Note that the orange cluster in motifs V and VIII relied, to a large extent, on the presence of external inputs to the LTS population; the area of the orange cluster was reduced markedly in motif IV (without external LTS drive) compared to motif V (with external LTS drive), with a similar effect visible for motifs VII and VIII, respectively (see Fig. 3). Motifs IV and VII only showed the low-frequency population activity and bursting typical for the orange cluster for a small set of input conditions, instead yielding stable PING oscillations for most input conditions. Our simulations therefore suggest that disinhibition-based circuits can switch between stable gamma oscillations to bursting behavior by increasing the drive to the LTS population. A demonstration of this principle is shown in Fig. 6g for the input conditions used in example 1 (Fig. 8b).

Are the RS-LTS dynamics in the three-cell-type motifs caused by the same mechanism as in motif II? The 4 three-cell-type motifs shared two characteristics with each other: (1) LTS cell received local excitation from the RS cells and (2) LTS cells inhibited the FS, but not the RS population. This connection pattern created local disinhibition of the RS cells through suppression of the inhibitory FS population. This resulted in reduced firing of FS cells, while stimulating RS and LTS firing (Fig. 5d). For low drive to the RS cells (and insufficient drive to the FS cells) in motifs IV, V, VII and VIII, this suppressed the activity in the FS population. Note that in these motifs, no direct inhibition of either the RS or LTS populations was present, as the FS cells were inactive (see Fig. 5d) and there was no connection from the LTS cells to the RS population, or between LTS cells. The lack of direct inhibition in the three-cell-type motifs shows that the oscillation in the orange cluster cannot result from an ING or PING like mechanism. Instead, the oscillation was caused by adaptation in the cell populations, captured in the model by an increase in the $U$ variable (see Methods), which truncated spiking activity in the RS population, in turn shutting down the activity in the LTS population. Conversely, in motif II inhibition of RS cells by the LTS population was present, and this difference could explain the increased oscillation frequency found in motif II (beta range) compared to the three-cell-type motifs included in the orange cluster (theta range), as build-up of this direct inhibition of the RS cells likely shortened the active period of this population.

In summary, the orange cluster is characterized by slow oscillations and burst firing in the RS and LTS populations, which is expected to switch to PING oscillations when the LTS drive is reduced. In the three-cell-type motifs, the oscillation is mediated by an absence of FS activity, allowing for burst firing and the slow buildup of adaptation in the other cell types.

### Three-cell-type motifs show frequency steps and stable asynchrony

In addition to new patterns of oscillatory dynamics, the clustering results for the three-cell-type motifs shown in Fig. 3e also make predictions about the way the motifs respond to changing inputs, some of which have already been discussed in the previous sections. Here, we take a closer look at the resilience of the circuit behaviors to changes in RS and FS inputs.

The introduction of the third cell type allowed the circuit to oscillate at low frequencies that are not observed in RS-FS circuits. As a result, it markedly increased the frequency range that can be achieved by the circuit under physiological conditions, from high beta to high gamma for the RS-FS circuit, to anywhere between theta and high gamma for the three-cell type circuit. As we have shown, these oscillations are caused by distinct mechanisms. This raises the question how the transitions between frequencies and mechanisms occur in the three-cell type circuit: are these transitions smooth and gradual, or abrupt and all-or-none?

As noted before, the frequency bands covered by the new yellow, orange and light blue clusters are concentrated in narrow frequency bands. As can be seen in the example motifs in Fig. 6f for the light blue cluster and in Fig. 8d for the orange cluster, the oscillation frequencies found for these clusters were largely independent from the level of inputs to the RS and FS populations. This behavior is markedly different from that of the purple PING cluster, where oscillation frequency gradually increased with RS and FS drive (compare Fig. 4d). The observed stability within the orange and light blue clusters has two potential consequences for the functional behavior of the oscillation: small or even medium changes in input to RS and FS are expected to have no impact on the oscillation for most input conditions, making it resilient to perturbations. However, when a similarly small change in RS or FS drive moves the circuit into a different cluster, the behavior of the circuit changes dramatically. For example, consider increasing the RS external drive in 1-pixel steps toward example 2 in Fig. 8. For the first few steps, the circuit dynamics appears unchanged, but when the boundary between orange and purple is approached, the circuit starts to briefly switch between an 8 Hz oscillation with high burst fraction and a sparse 40 Hz oscillation in the span of 2 input steps (Fig. 12c in Appendix III), after which the 40 Hz PING oscillation becomes the stable state. A similar behavior is seen for transition to the green cluster (Fig. 12d in Appendix III). This suggests that the introduction of the slow oscillations leads to sharp frequency steps, where small changes in drive to the RS or FS populations can lead to large changes in oscillation frequency.

We also asked whether the introduction of the second interneuron type affected the overall level of synchronization that is observed in the circuit across input conditions. For RS-FS circuit in motif I, 20.0% of input conditions led to low levels of synchronization (dark blue cluster). In most three-cell-type motifs, particularly those with local excitation to the LTS cells, this fraction remained similar (avg: 18.5%; range: 11.6–31.8%). However, in the motifs with only external LTS excitation (top row in Fig. 3e) the fraction of asynchronous behavior increased substantially (avg: 40.9%; range: 26.3–71.9%). This was particularly striking in motifs XV (45.6%) and XVIII (71.9%), where the substantial part of the tested input conditions lead to asynchrony and only very high input strengths produced oscillatory behavior. As such, the three-cell-type motifs cannot only stabilize oscillation frequencies, it can also result in stable asynchrony. Furthermore, as we pointed out before, the motifs in the top row of Fig. 3e can effectively be switched to the behavior of motif I by reduction in the LTS drive, suggesting that switches between asynchrony and synchronous PING oscillations in these circuits can be achieved by controlling the input to the LTS population.

Our modeling results therefore predict that the addition of LTS cells allows for oscillations at lower frequencies as well as asynchronous states, that are stable across many input conditions. In addition to this, depending on the circuit architecture, it also allows for sharp and substantial changes in network state, either between oscillation frequencies, or between asynchronous and synchronous states. In the model, these switches between states are controlled by changes in inputs to one of the cell types and these predicted changes are therefore both physiologically feasible and testable using optogenetic techniques.

## Discussion

In this study, we set out to provide a comprehensive characterization of the population dynamics of the circuits consisting of regular spiking pyramidal cells, fast-spiking PV cells and low-threshold spiking SOM neurons. We identified 18 possible circuit motifs with these three cell types (see Fig. 2), consisting of different connection patterns between the cell types and different input regimes for the SOM cells. We compared the behavior of these circuit motifs to that of two reference circuit motifs consisting of two cell types: RS-FS and RS-LTS. We used a *k*-means clustering approach and information criteria to identify 6 different groups of population dynamics across the 20 circuit motifs. Aside from asynchronous behavior, the motifs produced 5 distinct oscillatory behaviors, among which the previously identified PING and ING mechanisms. In addition to these well-known gamma oscillations, the three-cell-type motifs produced stable beta oscillations, theta-nested PING gamma oscillations, beta-nested ING gamma oscillations, as well as theta oscillations with burst firing. It is important to note that the model did not receive synchronized input to any of the cell types, and the oscillations were therefore spontaneously generated by the circuit. The modeling results provide important insights into the generative mechanisms behind three oscillations in physiologically relevant frequency bands and aid the functional interpretation of circuit behaviors recorded in neural tissue.

### k-means clustering allows for the identification of network signatures

A comprehensive comparison of 20 circuit motifs, as presented here, can be challenging due to the large number of datapoints and a priori unknown number and type of network dynamics. Here, we used a *k*-means clustering approach to categorize the dynamics into a small number of behaviors, in a data-driven way. The use of the Calinski–Harabasz index allowed us to determine the number of clusters based on the information in the data. It is important to note that this method did rely on two other choices that were made a priori: (1) the single unit and network dynamics descriptors that were included in the clustering algorithm (such as firing rate, peak frequency and burst fraction) and (2) the relative weighting of these characteristics (here, all set to 1). It is conceivable that including a different number or other descriptors, or changing their relative weights, would lead to merging or splitting of clusters, by emphasizing specific network dynamics. We validated our choices by applying the clustering approach to the two well-known motifs I and II and showed it correctly identified previously established circuit dynamics.

### Predictions for experiments using optogenetic stimulation and silencing

In this study, we included a wide range of input conditions for RS, FS and LTS cells. The model therefore allows us to predict the outcomes of a wide range of experiments. Our results suggest that LFP and spike recordings, combined with stimulation or inhibition of individual cell types, can be used to identify potential circuit motifs in vitro and in vivo. Although in the model none of the oscillatory behaviors were unique to a single circuit motif, these behaviors were indicative of subgroups of motifs. As a result, the model predicts that the number of possible circuit motifs can be narrowed down based on LFP and spike data alone. Furthermore, the model allows us to predict the outcomes of optogenetic stimulation for specific circuit motifs. Optogenetic stimulation of RS and FS cells can be seen as an increase along the vertical, respectively, horizontal axis of the plots in Figs. 3, 4, 5, 6, 7 and 8, while stimulation or reduction in LTS activity is captured by moving between the different rows of motifs in Figs. 2 and 3. Some of the predictions from the model are listed below:

- Stable beta oscillations occur across a wide range of three-cell-type motifs. These oscillations are ‘stable’ in the sense that changes to RS, FS or LTS drive are not likely to have a large impact on the power or frequency of the oscillation. Only a strong increase in FS drive is expected to affect the oscillation and is expected to switch the system to a high gamma-band ING oscillation, or to a beta-nested gamma oscillation.
- For motifs IX and XII, which are characterized by FS-LTS inhibition, with only external drive to the LTS population, the model predicts theta-nested gamma activity. It is further predicted that this pattern of activity changes to PING oscillations when the drive to the LTS cells is reduced.
- The model predicts that burst firing of individual cells (in the absence of intrinsic bursting behavior) depends on disinhibition of RS cells by LTS-to-FS connections, without local excitation of the LTS cells. Inhibiting the LTS cells in this motif is expected to reduce burst firing, allowing gamma oscillations to appear. Conversely, for motifs X and XI, where the LTS directly inhibits RS cells, inhibition of the LTS population could actually induce bursting, by moving these circuits closer to the setup in motifs VII and VIII.
- Stable asynchrony is also a predicted outcome of the three-cell-type motif. This behavior specifically occurred with feedforward inhibition where LTS cells only received external drive. In these cases, the model predicts that silencing of the LTS cells would lead to the appearance of a PING oscillation.

These predictions are based on the tonic activation of the individual cell types. Optogenetics is also well-suited to provide transient or periodic stimulation, allowing for the probing of resonance and entrainment in the circuit. Extending the modeling work presented here with periodic stimulation has the potential to produce even more comprehensive and detailed predictions that could aid the identification of specific motifs in neural tissue (Tiesinga 2012; Herrmann et al. 2016).

### Mechanisms for the generation of theta–gamma oscillations

The three-cell-type motif produced several oscillatory behaviors that are not present in circuits with RS and FS cells alone: theta–gamma oscillations, beta-nested gamma oscillations, beta oscillations and theta-bursting. The theta–gamma rhythm has been of particular interest in recent years due to its proposed mechanistic link to hippocampus-dependent spatial navigation and memory formation and its role in working memory maintenance (Düzel et al. 2010). The origin of theta oscillations remains a topic of study, and it is likely that the hippocampal network contains more than one theta-generative mechanism (Colgin 2016). One possible source of theta generation is thought to depend on subthreshold memory potential resonance mediated by h-currents. When O-LM cells were modeled with h-currents, spontaneous theta and theta-nested gamma oscillations could be generated (i.e., without external oscillatory current), but this critically relied on the presence of mutual inhibitory connections with fast-spiking interneurons (Rotstein et al. 2005). Theta–gamma oscillations were also demonstrated in a model network of PV and O-LM cells without h-currents, although these oscillations were less stable (White et al. 2000). This is echoed by modeling work, suggesting that the theta rhythm (Ferguson et al. 2017) as well as the theta-nested gamma signature (Pastoll et al. 2013) can be generated in the absence of h-currents when the circuit allows for local feedback inhibition. h-currents were also not necessary for theta–gamma oscillations in a more detailed model with RS, FS and LTS cells (Vierling-Claassen et al. 2010). Similarly, in a highly complex and detailed model of the hippocampal subregion CA1, output from PV cells was identified as crucial to the spontaneous theta-nested gamma oscillations produced in the model (Bezaire et al. 2016). Interestingly, this complex model showed that output from O-LM SOM-expressing cells to surrounding cells was not necessary for generating theta oscillations, although outputs from another dendrite-targeting cell type, neurogliaform cells, were found to be essential. The authors identified the slow synaptic dynamics of the neurogliaform cells as the essential element of contribution to the theta rhythm, similar to the findings for SOM-cells by (Vierling-Claassen et al. 2010). In line with the findings presented here, both studies also identified the FS-to-LTS connections as an essential ingredient for combined theta and gamma oscillations. Our results are based on LTS neuron models that implicitly incorporate an effect similar to that of h-currents, through the coupling between the two variables of the model. The theta-nested gamma oscillations relied on this coupling in the results presented here, but additional simulations shown in Fig. 13 (Appendix III) suggest that this behavior can also be achieved by introducing long synaptic decay time constants for the LTS projections. Our results therefore align with these previous studies, but extend them by demonstrating that theta-nested gamma oscillations can (1) be spontaneously generated in a network of simple point neuron models; and (2) be generated by two different implementations, namely h-currents and long synaptic decay time constants, in an otherwise identical network. Studying the similarities and differences between these implementations in detail is beyond the scope of this study.

### Mechanisms for the generation of beta oscillations

The rhythm that was most commonly produced by three-cell-type motifs was the beta oscillation, with stable frequencies between 20 and 30 Hz across a wide range of input conditions. Beta oscillations are a prominent part of cortical electrophysiological recordings, but were originally mostly linked to sensorimotor activity and motor-preparation. They have since been associated with a wide range of cortical regions and functional domains (for reviews see: Engel & Fries, 2010; Spitzer & Haegens, 2017). Experimental work has led to several proposed generative mechanisms. It has been suggested that beta oscillations can be generated in the same way as gamma oscillations in RS-FS circuits (through ‘INB’ and ‘PINB’ network mechanisms), but this relies on a substantially reduced GABA_A_ synaptic decay time constant (Jensen et al. 2005). Unless two or more PV cell populations with distinctly different GABA_A_ time constants can be identified, such a mechanism is unlikely to drive beta oscillations that spatially co-existing with faster gamma oscillations. Others have suggested that beta oscillations in motor areas are in fact independent of GABA_A_ receptors (Roopun et al. 2006). An alternative model for the generation of short periods of beta activity was proposed more recently, relying on the coincident input to apical and proximal dendrites of pyramidal cells (Sherman et al. 2016). Furthermore, it was shown that intrinsic bursting cells can produce spontaneous beta oscillations when their axons contain M-currents (Roopun et al. 2006; Kramer et al. 2008). The M-current builds up through burst spiking and, through hyperpolarization, prevents further spiking, with the decay time constant determining the period of these beta oscillations. When these intrinsic bursting cells were combined with RS, FS and LTS cells, the model circuit alternated between slow gamma, fast beta and a ‘period-concatenated’ slow beta, which period was the sum of the other two rhythms (Kramer et al. 2008). It is unclear whether this mechanism extends to other areas than motor cortex. Our modeling results show that the faster beta oscillation can in fact be obtained without either intrinsic bursting cells, or h- and M-currents, and can switch to gamma oscillations through changes in drive to the RS, FS and/or LTS cell populations. In line with experimental data, the model did not rely on GABA-mediated outputs from FS cells to other FS or RS cells.

### Burst firing during theta and beta oscillations

Furthermore, the model was able to generate burst spiking, without intrinsically bursting cells present in the circuit. The model also did not include dendritic mechanisms such as calcium-spikes and back-propagating action potentials in the RS cell population, which were demonstrated to mediate burst firing in layer 5 pyramidal cells (Larkum et al. 1999). In the model, burst firing was found for both RS and LTS in the orange cluster. This was associated with strong theta range oscillations. In addition, the burst fraction was high for LTS cells in the light blue cluster, where oscillations predominantly fell in the beta frequency range. These behaviors align with experimental data from monkey PFC, where increased burst firing in both excitatory and inhibitory populations was associated with increased theta power (Voloh and Womelsdorf 2017), while beta power changes were linked mostly to bursting in the inhibitory cells (Womelsdorf et al. 2014a; Voloh and Womelsdorf 2017). These findings depended on the attentional state of the animal, suggesting that the relationship between oscillations and (the recruitment of) burst firing is transient and dynamic. Our model predicts that such changes can be mediated by changes in drive to the local LTS population, a prediction that can directly be tested using optogenetic suppression of LTS firing in circuits with high burst firing. Further work is needed to assess the role of bursting cells and dendritic mechanisms in the generation of theta and beta oscillations in cortical tissue. While our work does not currently allow for a direct comparison of circuits with and without intrinsic bursting mechanisms, it does demonstrate that theta- and beta-associated bursting behavior can be obtained through circuit interactions alone, stressing the need for establishing both sufficiency as well as necessity of intrinsic bursting in future experimental studies.

### Model assumptions

This study provides a first step into the characterization of neural circuit dynamics of RS-FS-LTS motifs. Several assumptions and simplifications were made to make the comprehensive approach taken here feasible. For example, it was shown experimentally that interneurons of the same type form gap junctions with each other (Gibson et al. 1999), which were not included in the model. Gap junctions have the potential to synchronize activity of cells within a population directly and therefore can enhance their impact on downstream targets. Further modeling work is needed to determine the extent to which this affects the oscillatory dynamics presented here. We also did not include short-term facilitation and depression of chemical synaptic inputs in the model. The characteristics of synaptic facilitation and depression have been shown to differ between PV and SOM cells: Excitatory synapses onto SOM cells have been shown to facilitate, i.e., show an increase in the evoked excitatory post-synaptic potential (Reyes et al. 1998; Gibson et al. 1999; Beierlein et al. 2003). Most other synaptic connections, including the LTS-to-RS connections, show depression with repeated stimulation (Gibson et al. 1999; Beierlein et al. 2003), although recordings in hippocampus have suggested a more complex picture, with mixed facilitation and depression within cell type pairs (English et al. 2017). It was previously shown that facilitation of RS-to-LTS connections can give rise to slow oscillations (delta or low theta band) in a rate-based model (Hayut et al. 2011), i.e., without relying on spike timing. It is conceivable that short-term facilitation and depression interact with the oscillation-generating mechanisms described here. Future work will have to establish the form this interaction takes and to what extent this plays a role in physiological conditions.

**Fig. 9:**
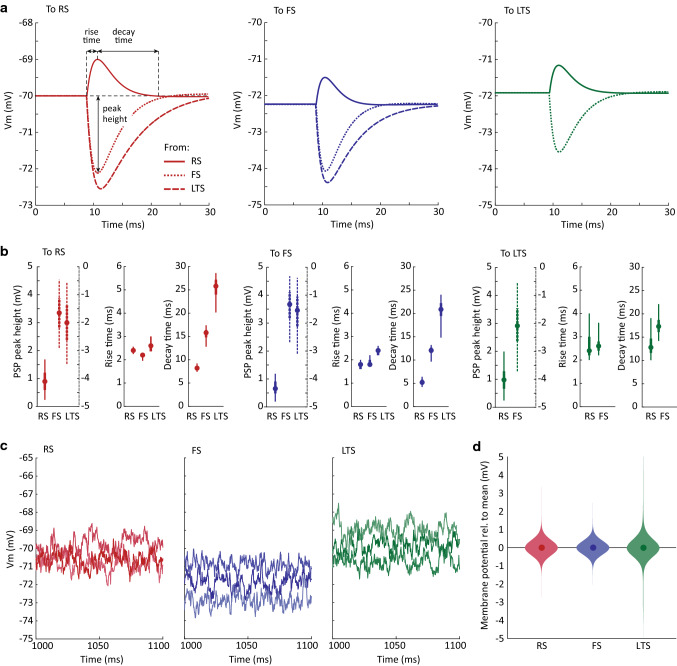
Characterization of synaptic and noise currents. **a**: Example post-synaptic potentials for RS (red), FS (blue) and LTS (green) cells, after receiving a single pre-synaptic action potential from either RS (solid), FS (dotted), or LTS (dashed) cells; **b**: Distributions of peak height (left), rise time (middle) and decay time (right) for post-synaptic potentials (PSP) as defined in a, for RS (red), FS (blue) and LTS (green) cells after receiving a single pre-synaptic action potential from the cell type giving on the * x*-axis. Note that the peak heights from RS cells correspond to the left-hand side * y*-axis, while inputs from FS and LTS cells correspond to the right-hand side *y*-axis. Distributions show data from 100 cells and 100 different synapses. Box plots indicate the 5, 25, 50 (circles), 75 and 95% boundaries; **c**: Example membrane potentials for each of the cell types receiving only noise current ${\text{I}}_{{{\text{noise}}}}$ (i.e., with no synaptic inputs and no background inputs ${\text{I}}_{{{\text{bg}}}}$); **d**: Membrane potential distributions caused by white noise current ${\text{I}}_{{\text{t}}}$ in each of the three-cell types relative to the mean membrane potential. Distributions show data from all cells in the network, i.e., N_RS_ = 800, N_FS_ = 100 and N_LTS_ = 100

**Fig. 10:**
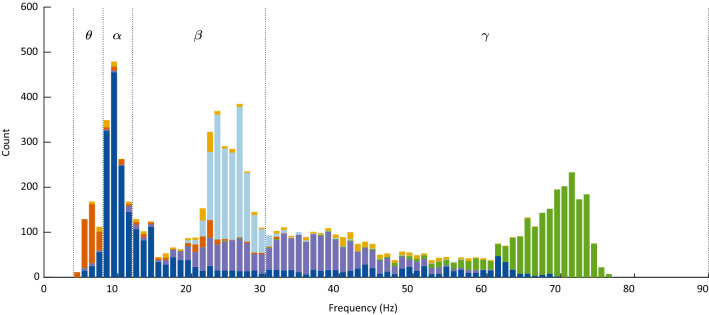
Distribution of average peak frequencies across all simulations per cluster. Color indicates the cluster, using the same color convention as in Figs. 3, 4 and 5. Dotted lines indicate boundaries of traditionally recognized frequency bands in electrophysiological recordings; theta: 4–8 Hz, alpha: 8–12 Hz; beta: 12–30 Hz and gamma: 30–90 Hz. The observed frequencies largely fall within these bands, although for the simulations, the alpha band seems to extend to approximately 15 Hz

**Fig. 11:**
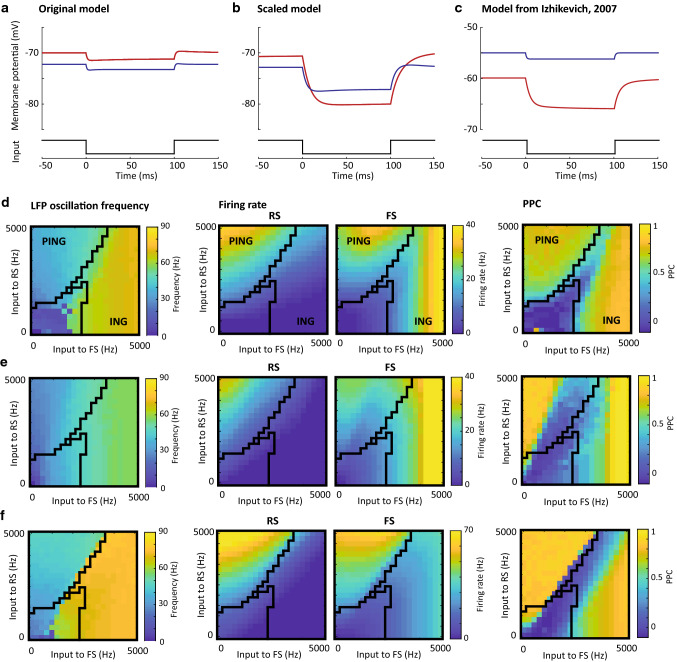
Examples of membrane time constant changes for RS and FS cells in Motif I. Shown are a single seed for three different models: **a & d:** the original model (corresponding to data in Fig. 4); **b & e:** a scaled version of the original model, in which the model equations were multiplied with a factor 0.1 for RS cells and 0.2 for FS cells, in order to substantially extent the membrane time constants; and **c & f:** the RS and FS models as published in (Izhikevich 2007). **a-c:** Example neuron models receiving a 100 ms block current for each of the three model types, demonstrating the differences in membrane time constant between model types and neuron types. RS cells are shown in red, FS cells in blue. **d-f:** Oscillation frequency in the LFP (left), firing rates of the RS and FS cells (middle, note the different firing rate scale in f) and the pairwise phase consistency (PPC, right) for each of the three model types. The different model types produce different firing rates and oscillation frequencies, but produce qualitatively similar network dynamics: a PING oscillation for low FS inputs and an ING oscillation for high FS inputs. For reference, black outlines represent the cluster borders as determined on the averaged data from the original model and shown in Fig. 4

**Fig. 12:**
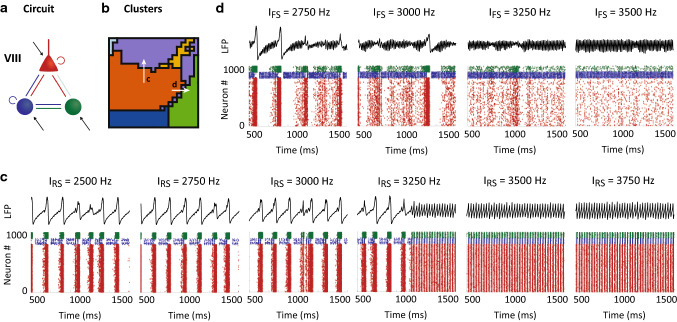
Examples of transitions between oscillatory states in motif VIII. **a**: Schematic of the circuit motif; **b**: Clustering result for this motif (repeated from Fig. 3 and 8). Arrows indicate the input conditions shown in panels c and d; **c & d:** Subsequent input conditions (left to right) leading to transitions from the orange cluster to the purple PING cluster **(c)** and the green ING cluster **(d)**. Top rows show the model LFP and bottom rows the raster plots, with RS cells in red, FS cells in blue and LTS cells in green. Both transitions are characterized by switches over time between the two cluster states when the input condition is close to the cluster’s boundary, due to noise in the inputs. Closer to the boundary, these switches become longer in duration

**Fig. 13:**
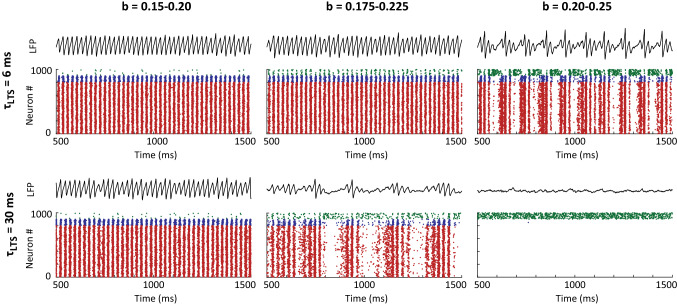
Examples of *b* and ${\tau }_{{{\text{pre}}}}$ parameter changes for the LTS cells in the yellow cluster of Motif IX, corresponding to example 1 in Fig. 7. Columns represent different b parameters for the LTS cell models, which represents the coupling of the membrane potential variable ${V}$ with the slow feedback variable ${U}$. The right column representing the value range used for the LTS cells throughout the remainder of the manuscript and the left column using the values equal to those used for the FS cells. The rows represent two different values of ${\tau }_{{{\text{pre}}}}$ used for the LTS synapses, with the value in the top row used in the remainder of the paper. Reducing the coupling of the ${V}$ and ${U}$ parameters switched the theta–gamma coupling to a gamma state when the synaptic time constant was short, but this could be rescued, at intermediate values, by increasing the synaptic time constant

**Fig. 14:**
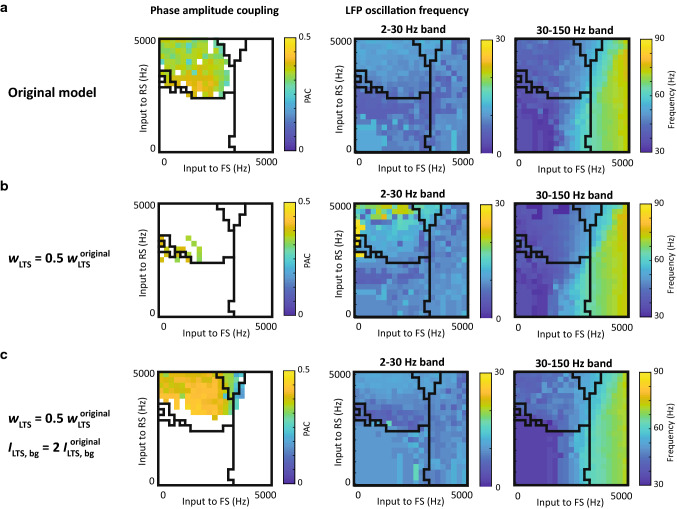
Examples of ${w}_{{{\text{LTS}}}}$ and ${I}_{{{\text{bg}}}}$ parameter changes for the LTS cells in the yellow cluster of Motif IX. **a**: Example run for the original model, for which the average across 10 seeds is shown in Fig. 7. Shown is the Phase-Amplitude Coupling (left) and the peak oscillation frequencies in the low (2–30 Hz) and high (30–150 Hz) frequency bands. **b**: As in a, but with the synaptic strength ${w}_{{{\text{LTS}}}}$ from the LTS cells to other cells halved. With weaker synapses from LTS cells, the area of phase-amplitude coupling is markedly reduced. **c**: This effect can be rescued by increasing the drive to the LTS cells, as shown here, where the background input to the LTS population, ${I}_{{{\text{bg}}}}$, was doubled. Black outlines represent the cluster borders as determined on the averaged data from the original model and shown in Fig. 7c

Several assumptions and simplifications had to be made in the model about the connections between the cell populations. Connections in neural circuits are far from uniform, depending on cell type and brain area (Silberberg and Markram 2007; Lee et al. 2013; Pfeffer et al. 2013; Jiang et al. 2013), but also on cortical layer (Yoshimura et al. 2005) and on downstream projections (Brown and Hestrin 2009). On small spatial scales, neurons are often densely connected, with density of connections falling off rapidly within a few hundred micrometers (Hellwig 2000). As oscillations, particularly at lower frequencies, are generally thought to span these larger spatial distances, our aim was to represent a network of at least several hundred micrometers with the model. However, to make simulation of a large number of circuit motifs and input regimes feasible, we simulated a relatively small number of cells, while maintaining the connection patterns seen when considering larger spatial scales. This is likely to result in an underestimation of the influence of local RS cells on the interneuron populations, which was compensated by relatively high, but random, input spike rates to all cell types. We also used relatively strong synaptic connections, to compensate for the lower number of neurons in the simulation. Given our interest in the effect of adding LTS cells to the well-known RS-FS circuit motifs, we opted for strong synaptic connections from LTS cells compared to RS and FS connections (compare Fig. 9a and b in Appendix I with results from, for example (Beierlein et al. 2003; Silberberg and Markram 2007; Pfeffer et al. 2013)). These stronger connections were aimed to mimic the input-gating effect that this neuron type is thought to have by inhibiting the apical dendrite, an effect which cannot directly be incorporated in our simplified point neuron model. Further work is needed to identify if, and if so, how these simplifications affect the presented results.

Another simplification lies in the use of relatively broad neuron categories, regular spiking, fast-spiking and low-threshold spiking cells, where many subgroups of these cell types exist in the brain. Interneurons in particular are very diverse, and efforts to classify PV and SOM cells are ongoing (Markram et al. 2004; Kepecs and Fishell 2014). Calcium-binding proteins, such as PV, and neuropeptides, such as SOM, are not universally unique identifiers and usually span several classic interneuron cell types (DeFelipe 1993; Markram et al. 2004). Detailed knowledge of the biophysical and morphological diversity within these cell types, as recently pioneered in (Gouwens et al. 2020), is needed to allow for more detailed models. Similarly, connectivity preferences of the difference subgroups need to be established, before a full assessment can be made of the impact this diversity has on the results presented here and in other studies using the same categories. Given more detailed neuron categories, future modeling work can also establish and/or predict in more detail, which neuron-specific characteristics are sufficient and required for generating the network dynamics demonstrated here and to what extent these characteristics are unique to PV and SOM cells.

### Possible roles of VIP neurons

In addition to within-group diversity, we also excluded several other known interneuron types that do not express PV or SOM. After PV and SOM cells, the most prominent interneuron type are the VIP neurons. VIP cells are thought to mostly project to SOM cells, while showing virtually no connections onto other VIP cells (Pfeffer et al. 2013). This results in a strong disinhibitory pathway, where activation of VIP cells leads to relief from inhibition for downstream PV and pyramidal cells (Pi et al. 2013). A powerful computational function of VIP cells could be to influence output selectivity, by disinhibiting PV cells and hence increased somatic inhibition in the local pyramidal cell population (Yang et al. 2016). Many examples of VIP-mediated disinhibition have been demonstrated recently (for example: Hertäg and Sprekeler 2019), but the influence on oscillatory mechanisms remains unclear. In the context of the model analyzed here, VIP cells could be considered, through its predominant projections to the LTS population, as a switch between high SOM drive and low SOM drive, i.e., between the first row of Fig. 3e (external drive) and motif I in Fig. 3a (no SOM drive), or between the third and the second row of Fig. 3e. Additional modeling work is required to develop these predictions further.

### Concluding remarks

In recent years, modeling of circuits with PV and SOM cells has led to interesting new insights into the many complex computations this circuit can sustain. Yet, more work is needed to comprehend the full scale of possible interactions seen in experimental work and to understand the often counterintuitive or contradictory results in optogenetics studies. Indeed, the model presented here demonstrates that even in a minimalistic model of Izhikevich point neurons, a wide variety of oscillatory behaviors can be generated. These results therefore stress the need for a comprehensive understanding of circuit dynamics, before appealing to more complex intrinsic mechanism in order to explain these oscillatory phenomena. Our findings provide a first step toward such a comprehensive description of circuits with PV and SOM interneurons.

## Data Availability

All code used to simulate and analyze the model presented here is available via https://github.com/marijeterwal/RS-FS-LTS-clustering.

## Appendix

### Appendix I: Characterization of input currents

See Fig. 9.

### Appendix II: Characterization of frequency bands

See Fig. 10.

### Appendix III: Exploration of model parameters

Motif I:

See Fig. 11.

Motif VIII:

See Fig. 12.

Motif IX:

See Figs. 13 and 14.
